# Facing Osteoporosis: Is Hormonal Therapy Losing an Opportunity to be Used? The Role of Gynecologists

**DOI:** 10.1055/s-0042-1760116

**Published:** 2022-12-29

**Authors:** Marcos Felipe Silva de Sá

**Affiliations:** 1 Editor –in – Chief RBGO

In the month of October, the World Day against Osteoporosis was celebrated and the date is dedicated to the global awareness of its prevention, diagnosis and treatment. This disease affects about 200 million people worldwide, causing more than 8.9 million fractures annually. 1 2 Globally, it affects 21.2% of women over 50 years of age. 3 Fractures caused by osteoporosis have great importance not only because of their high prevalence, but also because of their serious physical, psychosocial and financial consequences that affect both individuals and their families, the community and health systems.

Considering these demographic aspects of the disease, the Brazilian Federation of Gynecology and Obstetrics Associations – FEBRASO - joined other organizations and medical societies in the campaign, since climacteric women are among the population most at risk for osteoporosis, who nowadays correspond to a large percentage of patients in gynecology offices. According to surveys by DataFolha, one of the main institutes of public opinion in Brazil, eight in every ten women consider the specialty of Gynecology and Obstetrics as the most important for women's health in Brazil. 4 For brazilian women , Gynecologist-Obstetricians are considered as reference physicians, whether for treating problems of the specialty, as well as for counseling and guidance when they need healthcare in another medical specialty. 4 Thus, the gynecologist's office becomes one of the main entry points into the health system for women, and this professional should become a true sentinel in the screening for osteoporosis, seeking to identify women at risk for fractures. Therefore, gynecologists must be prepared for this task and also Also to refer the patient when the osteoporosis etiology requires joint care with another medical specialty since this disease has multiple origins and patients often require multidisciplinary follow-up. Thus the Competence Matrix for Medical Residency Programs in Gynecology and Obstetrics stablished by FEBRASGO emphasize the attention to climacteric women's health. 5 In addition, Febrasgo created the National Specialized Commission on Osteoporosis, which has been developing an excellent work, aiming to reinforce the fundamentals to approach patients at risk for osteoporosis. . With the active participation of members of that Commission, the Brazilian Osteoporosis Manual was recently launched, conceived as a practical guidelines for health professionalss. 6

Once the osteoporosis have been identified, it is essential that the physician offer to the patient all the information about the disease in order that she must be awared about the risks of suffering fracture. Thousands of women with this silent disease are unaware of these risks. Currently there are international consensus regards the necessity of the patients to change her lifestyle, adequate diet, use of calcium and vitamin D, physical exercise, avoiding alcoholic beverages, among others, in addition to supplementing with medication when necessary. Several options of pharmacological treatment are available on the market, such as Hormone Replacement Therapy (HT), Bisphosphonates (BP), Selective Estrogen Receptor Modulator (SERMs), Denosumab, Teriparatide and others still being studied. All options have advantages and disadvantages and the choice will depend on the professional's experience in dealing with each medication, considering its possible side effects and/or complications of its use. Since the disease has multiple triggering factors and patients are treated by different medical specialties, drug therapy has varied according to treatment protocols established by different specialty societies.

It is known that one of the main trigger for the development of osteoporosis in women is estrogen deficiency consequent to physiological or induced ovarian failure, which determines an increase in bone resorption that is not compensated by an equivalent increase in formation. 7 The medical literature has consistently and significantly shown that HT (encompassing both estrogenic therapy and estrogen-progestin therapy ) is indicated for climacteric women when they presented with vasomotor symptoms and genitourinary syndrome of menopause. Besides that, TH may be considered to be used to prevent bone loss and fragility fractures. 8 9 10 Estrogens have a positive effect on reducing the risk of fractures of the hip, vertebrae and other related fractures in postmenopausal women. It is the only therapy available with proven randomized clinical trials presenting effectiveness in reducing fractures, even in groups of women who do not have an identified risk for fractures or who have a T-score in the normal or osteopenic range in bone mineral densitometry (BMD). 11 Considering its well-known contraindications, 10 12 the HT in climacteric can be started in women at risk of fractures or osteoporosis before the age of 60 or within the first ten years after menopause (window of opportunity). There is an international consensus supported by influential Specialty Societies that indicate its use, evidently establishing individualized safety criteria for each patient. 9 HT would be the best choice for climacteric women in that period, because in addition to the undeniable benefits on bone mass, , patients have the opportunity to the additional benefits offered by HT, such as the prevention or abolition of hot flashes, , protection against genitourinary syndrome of menopause and its consequences on sexual health, positive effects on collagen and skin and significantly improving in sleep and quality of life. 10

Since HT is a routine prescription for gynecologists, it should obviously be the first choice for these patients, considering the extra benefits mentioned above. However, contrary to what is expected, an increasing use of BP has been observed as the first choice among gynecologists rather than HT, even for those climacteric patients considered within the window of opportunity who do not have any contraindication for its use.

Why gyneologists are missing this opportunity to prescribe HT? Several factors may be contributing to this behavior. The first to be cited would still be the impact of the Women's Health Initiative (WHI) study published two decades ago, which raised fears among physicians and patients about the risk of the association between HT and breast cancer and cardiovascular diseases. 13 It is known that many patients refuse to use HT for fear of breast cancer, often as a result of misinformation or because they receive distorted information from their own doctors , a common fact in current times, where social networks have negatively contributed to the dissemination of information from unqualified origin that reaches both, patients and healthcare professionals. It is important that the physician has up-to-date information on these topics , through reliable sources to better guide patients about the real risks of its use. The negative impact of the WHI study has been revised in recent publications as its original data have been reviewed in more detail. When subdividing patients by age groups and analyzing separately the effects of therapy with estrogen alone versus the estrogen-progestin combination the results show that the risks within the window of opportunity period are minimized, with the benefits of HT being greater than the risks of its use 14 . It should be noted that in the WHI study the mean age of evaluated patients was 63 years (including patients up to 79 years old), 13 therefore, well above the age currently suggested in the international literature for the introduction of HT. HT may be safe for a period of five years, which can be extended to ten years, depending on the patient's response to treatment, always under careful supervision of the attending physician. 10 The fact that hormone doses recommended nowadays are much lower compared to those used two decades ago should also be taken into account. There are different therapeutic schemes with proven cost-effectiveness and available in most countries including Brazil. 10 12 There is a worldwide trend towards its use by the non-oral route given the lower risks and side effects.

In their Medical Residency Program , gynecologists received guidance and practices for the use of HT, including training to deal with its side effects, especially the management of abnormal uterine bleeding, which are frequent and constitute additional difficulties for prescribers from other medical specialties who may assist these patients. Perhaps this is the main reason for non-gynecological specialists to choose therapies other than HT therapy and . in this sense, the gynecologist may have an advantage to safer prescribe the HT. It is worth adding that the possible risks of HT disappear quickly when its use is discontinued, unlike BP, which prolonged use, particularly beyond five years, compromises the bone structure and decreases its resistance, with the risk of significant adverse effects, since its residual effects may persist for several years after discontinuation. 15

Doctors specialists in Gynecology should reflect on this issue and, in order to avoid abuses in its prescription, it is good to remember that HT should not be recommended without a clear indication for its use and must be in accordance with the acceptance of the patient. and her priorities in terms of health as opposed to personal risks aiming her quality of life
